# Concrete Surface Crack Detection Algorithm Based on Improved YOLOv8

**DOI:** 10.3390/s24165252

**Published:** 2024-08-14

**Authors:** Xuwei Dong, Yang Liu, Jinpeng Dai

**Affiliations:** 1 Key Laboratory of Opto-Electronic Technology and Intelligent Control, Ministry of Education, Lanzhou Jiaotong University, Lanzhou 730070, China; dxw007@lzjtu.edu.cn (X.D.); 12221950@stu.lzjtu.edu.cn (Y.L.); 2 National and Provincial Joint Engineering Laboratory of Road & Bridge Disaster Prevention and Control, Lanzhou Jiaotong University, Lanzhou 730070, China

**Keywords:** concrete surface crack detection, YOLOv8, deep learning, LSKA, GSConv, ghost

## Abstract

Concrete surface crack detection is a critical research area for ensuring the safety of infrastructure, such as bridges, tunnels and nuclear power plants, and facilitating timely structural damage repair. Addressing issues in existing methods, such as high cost, lengthy processing times, low efficiency, poor effectiveness and difficulty in application on mobile terminals, this paper proposes an improved lightweight concrete surface crack detection algorithm, YOLOv8-Crack Detection (YOLOv8-CD), based on an improved YOLOv8. The algorithm integrates the strengths of visual attention networks (VANs) and Large Convolutional Attention (LCA) modules, introducing a Large Separable Kernel Attention (LSKA) module for extracting concrete surface crack and local feature information, adapted for features such as fracture susceptibility, large spans and slender shapes, thereby effectively emphasizing crack shapes. The Ghost module in the YOLOv8 backbone efficiently extracts essential information from original features at a minimal cost, enhancing feature extraction capability. Moreover, replacing the original convolution structure with GSConv in the neck network and employing the VoV-GSCSP module adapted for the YOLOv8 framework reduces floating-point operations during feature channel fusion, thereby lowering computational complexity whilst maintaining model accuracy. Experimental results on the RDD2022 and Wall Crack datasets demonstrate the improved algorithm increases in mAP50 by 15.2% and 12.3%, respectively, and in mAP50-95 by 22.7% and 17.2%, respectively, whilst achieving a reduced model computational load of only 7.9 × 10^9^, a decrease of 3.6%. The algorithm achieves a detection speed of 88 FPS, enabling real-time and accurate detection of concrete surface crack targets. Comparison with other mainstream object detection algorithms validates the effectiveness and superiority of the proposed approach.

## 1. Introduction

Since the 1990s, a large number of social and civil infrastructure, such as bridges, tunnels and nuclear power plants, have been constructed throughout China. Owing to its low cost, versatility and extensive use, concrete has become one of the most widely used materials in various types of infrastructure. However, concrete structures inevitably face crack problems caused by creep, shrinkage and loads, which may compromise their safety [1]. Moreover, as buildings age, they are increasingly affected by factors such as material aging, long-term loads, frequent and sudden natural disasters and human-induced damage, leading to varying degrees of crack damage in concrete structures. If not repaired promptly, these cracks may pose marked safety risks to concrete infrastructure and potentially threaten human life. Therefore, timely detection of concrete surface cracks has profound significance for preventing infrastructure damage and maintaining its safety.

Concrete crack detection methods primarily include traditional and deep learning methods. Traditional methods are based on crack detection using traditional digital image processing. The detection procedure based on image processing generally includes three stages: firstly, pre-processing crack images through denoising and filtering; secondly, binarization and morphological processing by setting thresholds; and thirdly, classification detection using classifiers. Abdel-Qader et al. [2] analyzed crack edge information using the Fourier and Hough transform algorithms and segmented cracks using the Canny operator to extract crack edge information. Salman et al. [3] designed an automatic crack digital image classification method using Gabor filters. Zhou et al. [4] proposed an automatic method for detecting cracks using frequency domain filtering and contour analysis of 3D laser range data. Vivekananthan et al. [5] combined grayscale discrimination and the Otsu method to successfully detect target cracks in different images. Zhu et al. [6] proposed a crack detection framework based on two-dimensional digital image correlation and a displacement-based robust crack detection method for concrete cracking phenomena, which evaluated their fracture performance.

In recent years, with the rapid development of deep learning technology, crack detection algorithms based on object detection techniques have become mainstream. Object detection techniques are classified into two- and single-stage algorithms. The current mainstream two-stage algorithms include R-CNN, Fast R-CNN and U-Net, to name a few. Rosso et al. [7] developed an artificial intelligence (AI)-based automatic road tunnel defects hierarchical classification framework to improve the efficiency of the indirect surveying method. Shahin et al. [8] constructed a new concrete crack detection model by combining ViT models with multiple image enhancement detectors. Chun et al. [9] proposed a method combining fully convolutional neural networks and semi-supervised learning to segment pavement crack images, but this method produced rough results that were prone to false detections. Ghosh et al. [10] improved the convolutional layer structure of U-Net using ResNet residual blocks, validating their performance on public datasets and achieving excellent results. Kang et al. [11] used an ensemble approach, incorporating the Faster R-CNN algorithm for faster regional convolutional neural networks to detect crack areas. Meng et al. [12] classified cracks, coarse segmentation and fine segmentation and extracted maximum widths using lightweight classification algorithms, lightweight segmentation algorithms, high-precision segmentation algorithms and crack width measurement algorithms to achieve an automatic real-time crack detection method based on drones. Another category is single-stage detection methods, which directly separate specific categories and regress boundaries. The single-stage object detection algorithms primarily include the You Only Look Once (YOLO) series and the Single Shot MultiBox Detector (SSD). Compared with single-stage algorithms, two-stage algorithms have certain advantages in detection accuracy, but their detection speed is much slower than single-stage algorithms. Therefore, single-stage algorithms are extra concerned with concrete surface crack detection. Chen et al. [13] introduced a deep learning framework, combining convolutional neural networks (CNNs) with naive Bayes‘ decision algorithms to detect boundary box cracks on underwater nuclear power station surfaces. Deng et al. [14] applied YOLOv2 for automatic crack detection in concrete, evaluating the robustness of the trained detector against interference by handwritten notes and finding that YOLOv2 can automatically locate a cracked bounding box from original images, even under handwriting interference. Liu et al. [15] proposed a bridge crack detection algorithm, R-YOLO v5, largely improving crack detection accuracy by incorporating attention mechanisms and optimizing loss functions. Wu et al. [16] proposed an improved YOLOv4 network using pruning techniques and EvoNorm-S0 structures, which better identified concrete cracks with several misleading targets. They found that whilst maintaining high accuracy, the model could correctly classify cracks at a faster computational speed. Ye et al. [17] proposed an improved YOLOv7 network and enhanced three different self-developed modules to better identify concrete cracks from several misleading targets, demonstrating effective detection of cracks of different sizes and robustness in validating images contaminated with various types and intensities of noise. Jiang et al. [18] optimized the YOLO-v3 and SSD algorithms using depth-separable convolution, inverse residual networks and linear bottleneck structures, finding that optimized detection accuracy increased by 3.25% and 4.04%, respectively.

As one of the representatives of single-stage object detection algorithms, the YOLO algorithm has received widespread attention from scholars since its inception. In recent years, with continuous optimization updates, the YOLO algorithm has achieved increasingly excellent results in object detection. In 2023, the Ultralytics team proposed YOLOv8, which not only boasts of high-precision recognition but also excellent real-time performance. The team further lightweighted the entire framework, rendering it extra suitable for concrete surface crack detection. Therefore, based on YOLOv8n, this paper optimizes the model to improve the accuracy of concrete surface crack detection, proposing a YOLOv8-CD algorithm for concrete surface crack detection. Firstly, by introducing the Large Separable Kernel Attention module [19], other abundant and fine characteristic information about cracks on concrete surfaces is captured, providing strong support for accurate crack identification. Secondly, to further enhance the feature extraction capabilities, the Ghost module [20] is integrated into the backbone network of YOLOv8, improving detection accuracy and optimizing overall model performance. Finally, in the neck network of the model, the GSConv is used to replace traditional convolutional structures and integrate the VoV-GSCSP module [21]. This method not only ensures model accuracy but also effectively reduces floating-point operations during feature channel fusion, further reducing model parameters and enhancing computational efficiency. Thus, the proposed algorithm in this paper can further strengthen the application of deep learning algorithms in concrete crack detection technology, offering ideas for deploying related detection algorithms to embedded rapid detection devices, improving the accuracy and efficiency of crack detection and providing technical support for the timely elimination of safety risks in concrete infrastructure.

## 2. Algorithms

### 2.1. The YOLOv8 Algorithm

The You Only Look Once (YOLO) algorithm was initially proposed by Joseph Redmon et al. in 2016. Unlike traditional object detection methods, YOLO adopts a novel approach by transforming the object detection task into a regression problem of a single neural network. It divides the image into multiple grids, predicts bounding boxes and object categories within each grid and utilizes the Non-Maximum Suppression (NMS) algorithm to eliminate overlapping bounding boxes, thus achieving real-time object detection.

The YOLO series algorithms represent notable advancements in the field of object detection. YOLOv1 [22] was the first single-stage object detection algorithm, achieving end-to-end prediction. YOLOv2 [23] introduced the Darknet-19 network, profoundly enhancing detection accuracy and speed. YOLOv3 [24] employed the deeper Darknet-53 network. YOLOv4 [25], proposed by Alexey Bochkovskiy et al., used the CSPDarknet53 network to improve performance. YOLOv5, introduced by Glenn Jocher, achieved remarkable results in many object detection tasks. YOLOv6 [26], developed by Meituan, focused on industrial applications. YOLOv7 [27], proposed by the YOLOv4 team in 2022, transformed object detection into a regression problem through convolutional neural networks and fully connected layers for prediction.

The YOLOv8 algorithm used in this paper was introduced by Ultralytics in 2023. It adopts a lightweight network structure whilst maintaining the efficiency and ease of use typical of the YOLO series, further enhancing algorithm performance and flexibility for tasks such as image classification, object detection and instance segmentation, supporting both CPU and GPU platforms. YOLOv8 introduces several innovations, including a new backbone network, Anchor-Free detection head and loss functions and deformable convolution DCNv3, significantly boosting performance. Additionally, the algorithm employs dual-path prediction and densely connected convolution networks, decomposing object detection into classification and localization subtasks. Then, it applies cascading and pyramid concepts to detect objects of different sizes.

**Figure 1:**
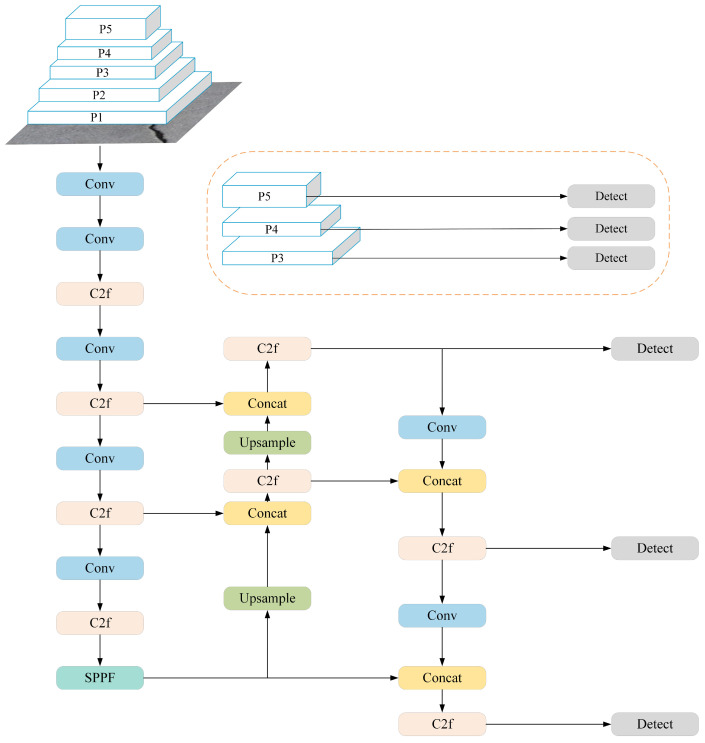
YOLOv8n network architecture.

**Figure 2:**
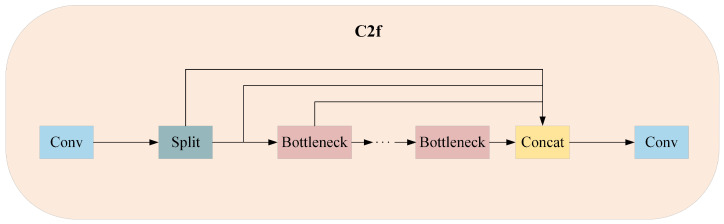
The C2f module of YOLOv8n.

**Figure 3:**
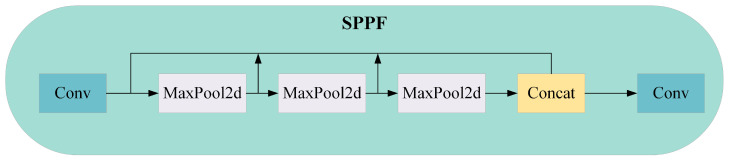
The SPPF module of YOLOv8n.

Figure 1 is the network structure of YOLOv8. The backbone consists of C2f and SPPF modules with the CSP concept, whilst the neck network of the backbone adopts the ELAN structure design principles from YOLOv7, replacing the C3 module of YOLOv5 with the more gradient flow of the C2f structure. As shown in Figure 2, the C2f structure enhances the feature fusion capability of convolutional neural networks, improves inference speed and achieves further lightweighting. Moreover, YOLOv8 uses the SPPF module from YOLOv5 architecture, as shown in Figure 3, which is a spatial pyramid pooling layer that expands the receptive field, facilitates local and global feature fusion and enriches feature information. The head section undergoes remarkable changes compared with YOLOv5, adopting the mainstream decoupled head structure and separating classification and detection heads. YOLOv8 departs from Anchor-Base methods to embrace Anchor-Free principles; on loss functions, it employs the BCE Loss for classification and the DFL Loss + CIOU Loss for regression. In terms of sample matching, YOLOv8 uses Task-Aligned Assigner instead of IOU matching or single-sided proportional allocation.

### 2.2. Problems with YOLOv8

When deploying the YOLO algorithm to mobile devices, the limited performance of mobile devices needs consideration. At the same time, the requirement for real-time detection of concrete surface cracks is high. Accordingly, this paper selects YOLOv8n, the minimum weight model algorithm of YOLOv8. However, in the actual detection process, YOLOv8n also has certain defects. Firstly, the YOLOv8n algorithm uses numerous standard convolutions and C2f modules, which improves the accuracy of the algorithm but reduces the running speed and increases the parameters of the model. Secondly, the detection scene changes quickly in mobile detection, and ensuring sufficient detection accuracy is difficult. Therefore, the YOLOv8n algorithm is not ideal for the detection and processing of concrete surface cracks, as it is prone to problems such as false detection and missing detection.

## 3. Improvements to YOLOv8

**Figure 4:**
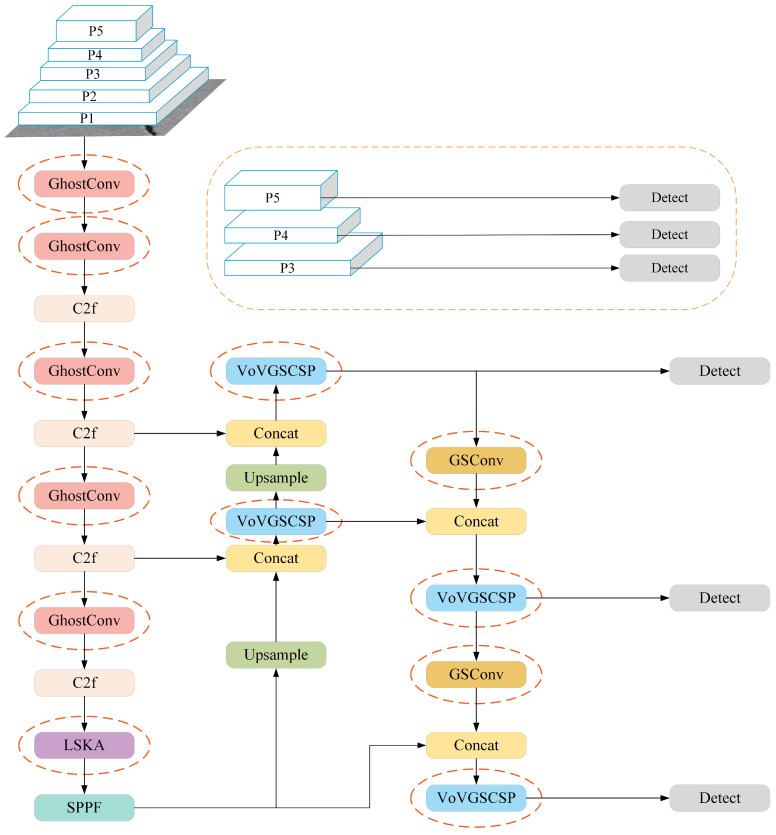
YOLOv8-CD network architecture.

To improve the accuracy of concrete crack detection, this paper proposes an improved detection network model, called YOLOv8-CD, based on YOLOv8n, as shown in Figure 4. Prior to the SPPF module in the YOLOv8n backbone network, the LSKA module, incorporating visual attention networks and a large convolutional attention, was embedded to accommodate features such as brittle cracking, large span and the elongation of crack objects. Additionally, the Ghost module was employed within the backbone network to extract necessary information from original features at a lower cost, thereby enhancing feature extraction capability. In the neck network, GSConv replaced the original convolution structure, and the VoV-GSCSP module was utilized to reduce floating-point operations during feature channel fusion and decrease model parameters whilst ensuring accuracy.

Additionally, this paper conducts detailed hyperparameter tuning for the YOLOv8-CD algorithm. Specific adjustments include determining an appropriate learning rate to ensure stable model convergence; selecting a suitable batch size to effectively utilize GPU memory and maintain training stability; adjusting the number of network layers and structure, based on the characteristics of the dataset, to fully extract and represent crack features; optimizing the size and ratio of anchor boxes to improve detection accuracy and recall rate; and adjusting L2 regularization parameters to prevent overfitting. These hyperparameter adjustments significantly improved the model’s detection accuracy and generalization capability on the dataset.

### 3.1. The LSKA Module

Owing to the complex and variable environments where concrete surface cracks occur, enhancing the model’s ability to represent crack damage features is crucial. In the YOLOv8 backbone network, an attention mechanism was integrated to locally enhance features, allowing the network to ignore irrelevant information interference and incorporate other valuable information into the fused feature maps. Traditional attention mechanisms, such as Squeeze and Excitation (SE) and the Convolutional Block Attention Module (CBAM), have notable limitations. SE attention focuses solely on inter-channel dependencies, consequently neglecting spatial features. Meanwhile, the CBAM introduces large-scale convolution kernels to extract spatial features but overlooks long-range dependency issues.

**Figure 5:**
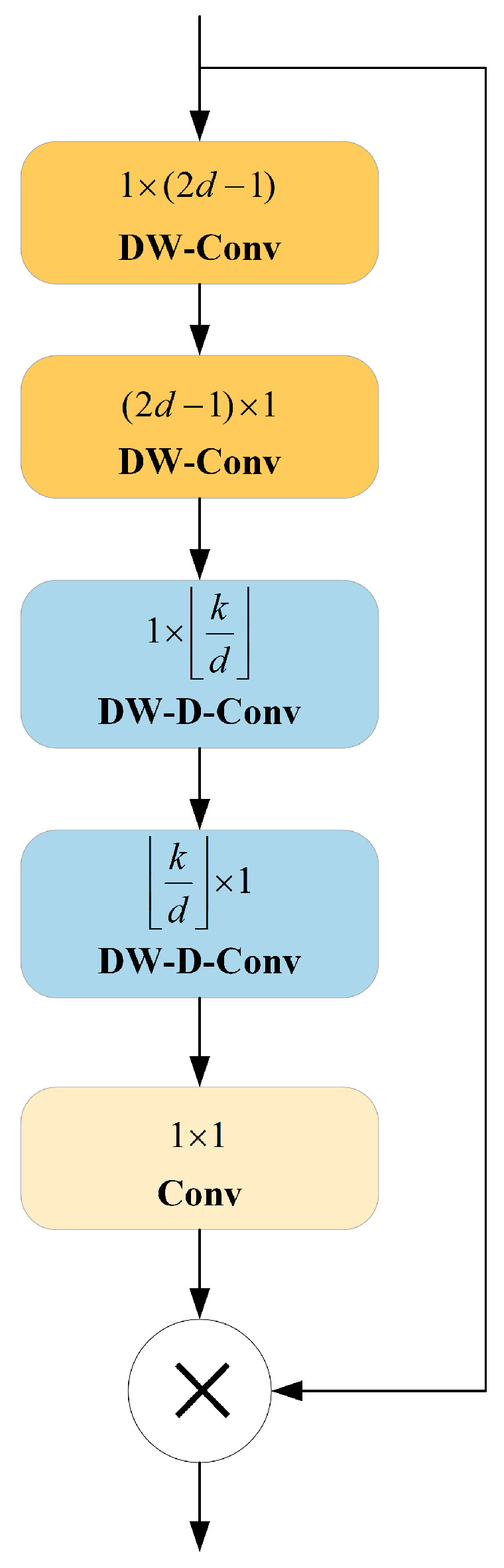
The LSKA module.

**Figure 6:**
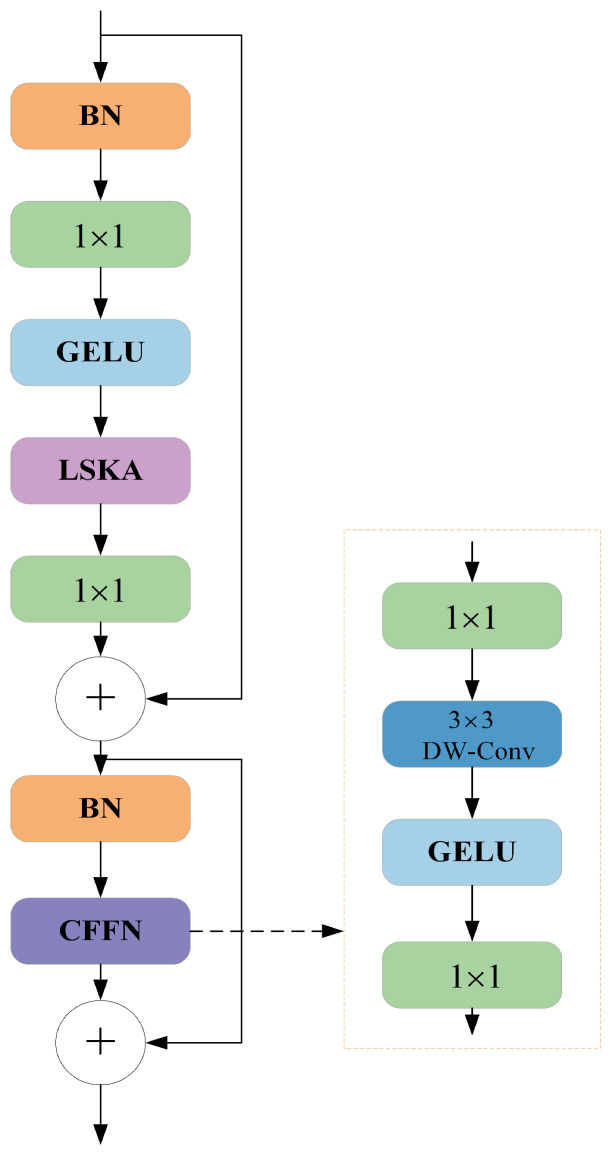
Block design of LSKA in VAN.

Large Separable Kernel Attention (LSKA) is an improved module for the application of the traditional Large Kernel Attention (LKA) module in visual attention networks. Traditional LKA modules have the disadvantages of high computational complexity and memory requirements when handling large convolution kernels. To mitigate these problems, LSKA employs oversized convolution kernels in the attention modules of VAN, as shown in Figure 5. It decomposes the two-dimensional convolution kernels of deep convolution layers into stacked horizontal and vertical one-dimensional kernels, achieving the goal of reducing computational complexity and memory usage. This improvement allows the model to maintain high performance whilst alleviating computational and memory burdens. In this paper, introducing LSKA further enhanced the detection accuracy and efficiency of the model. By optimizing network structure and attention mechanisms, the model better captured critical features in images, thereby improving the accuracy of object detection. Figure 6 shows the block design structure of LSKA within the visual attention network.

### 3.2. Ghost Module

**Figure 7:**
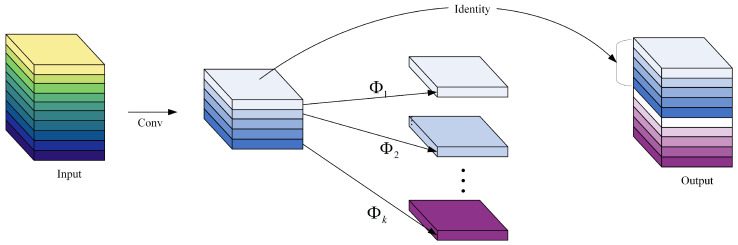
The Ghost module.

The conventional method for feature extraction involves convolving all channels of input feature maps with multiple convolution kernels. However, in deep networks, stacking numerous convolutional layers requires a substantial number of parameters and computations, resulting in many redundant feature maps. Therefore, several studies have proposed model compression methods, such as pruning, quantization and knowledge distillation [28,29,30]. Some problems, such as complicated model design and difficult training, exist despite their effectiveness in reducing parameters. Other approaches focus on optimizing network structures, such as MobileNet and ShuffleNet [31,32]. However, a 1 × 1 convolutional layer still consumes considerable memory and Giga floating-point operations per second (GFLOPs). Hence, acquiring essential feature maps is needed without complete convolutional operations. The Ghost module (in Figure 7) uses standard convolutions to obtain partial feature maps, linear operations to generate additional feature maps and, finally, concatenates these two sets of feature maps to a specified dimension, achieving other feature mappings with fewer parameters and computations.

The operation of generating n feature maps in any convolutional layer can be defined as follows:

(1) Y=X×f+b

where $X\in R^{c\times h\times w}$ is the input, c is the number of input channels and h and w are the height and width of the input feature map. $Y\in R^{h^{\prime }\times w^{\prime }\times n}$ is the output feature map with n channels, $h^{\prime }$ and $w^{\prime }$ are the height and width of the output feature map, $f\in R^{c\times k\times k\times n}$ is the convolutional filter of this layer with a kernel size of k·k and b is the bias term. The computation result of this convolution is as follows:

(2) $$n\cdot h^{\prime }\cdot w^{\prime }\cdot c\cdot k\cdot k$$

For the Ghost module, the first step is to obtain the intrinsic feature maps ($Y^{\prime }$) using conventional convolution, with a computational complexity of the following:

(3) $$Y^{\prime }=X\times f^{\prime }+b$$

Then, each channel of feature maps $Y^{\prime }$ is processed using operation $\varphi_{ij}$ to generate the Ghost feature maps $Y_{ij}$:

(4) $$y_{ij}=\varphi_{i,j}\left(y_{i}^{\prime }\right),\;\forall i=1,\ldots ,m,\;j=1,\ldots ,s$$

Finally, the intrinsic feature maps obtained in the first step are concatenated (identity concatenate) with the Ghost feature maps obtained in the second step to obtain the final result.

If the input tensor is c·h·w (input channels, feature map height and width), after one convolution, the output tensor is $n\cdot h^{\prime }\cdot w^{\prime }$ (output channels, feature map height and width). If the conventional convolution kernel size is k and the linear transformation convolution kernel size is d, after s times transformations, the computational complexity comparison between regular convolution and Ghost convolution operations is as follows:

(5) $$r_{s}=\frac{n\cdot h^{\prime }\cdot w^{\prime }\cdot c\cdot k\cdot k}{\frac{n}{s}\cdot h^{\prime }\cdot w^{\prime }\cdot c\cdot k\cdot k+\left(s-1\right)\cdot \frac{n}{s}\cdot h^{\prime }\cdot w^{\prime }\cdot d\cdot d}=\frac{c\cdot k\cdot k}{\frac{1}{s}\cdot c\cdot k\cdot k+\frac{s-1}{s}\cdot d\cdot d}\approx s$$

where n/s is the number of output channels after the first transformation; owing to the identity mapping, s−1 does not need to be calculated, but it is considered part of the second transformation. Therefore, the computational complexity of the Ghost module is approximately 1/s times that of regular convolution.

**Figure 8:**
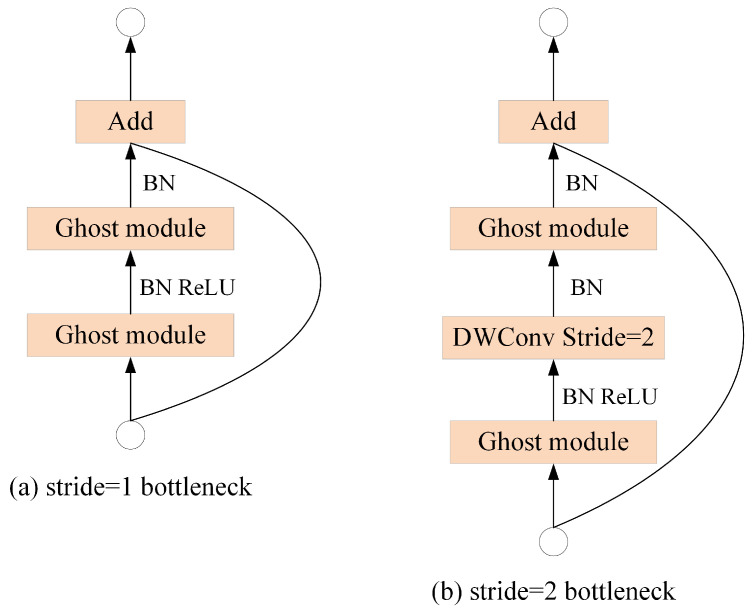
The Ghost Bottleneck module.

Taking advantage of the Ghost module, a Ghost Bottleneck is achieved specifically for CNNs. As shown in Figure 8, the Ghost Bottleneck resembles the basic residual blocks in ResNet [33], incorporating multiple convolutional layers.

This study enhances feature extraction capability by leveraging the Ghost module within the backbone network, extracting the necessary information from raw features at minimal cost. It simultaneously reduces the overall model size, thereby ensuring lightweight design without compromising accuracy.

### 3.3. GSConv and VoV-GSCSP Modules

Real-time performance is crucial for object detection, as it requires accurate identification of objects in images or videos within extremely short timeframes, providing relevant information such as their position and size. This scenario demands algorithms not only with efficient processing capabilities but also with rational optimization and configuration of hardware devices. However, lightweight models constructed from numerous depthwise separable convolution layers often fail to achieve sufficient accuracy. Therefore, this paper introduces a novel approach, GSConv, which maintains adequate accuracy whilst reducing model complexity.

**Figure 9:**
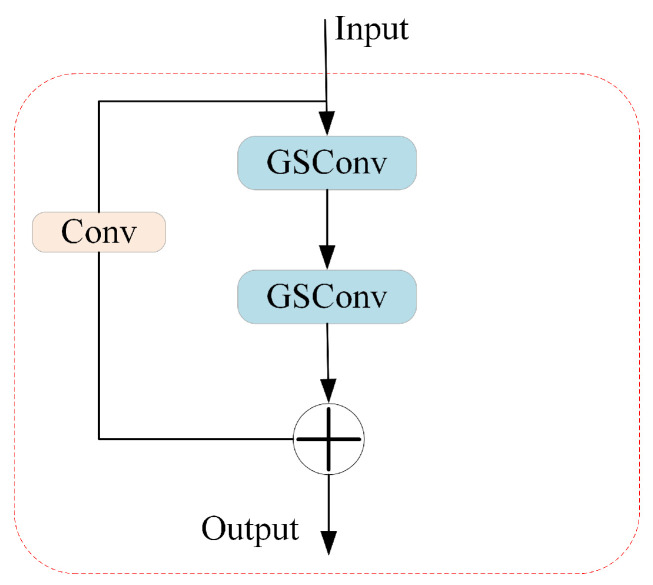
The GS Bottleneck module.

**Figure 10:**
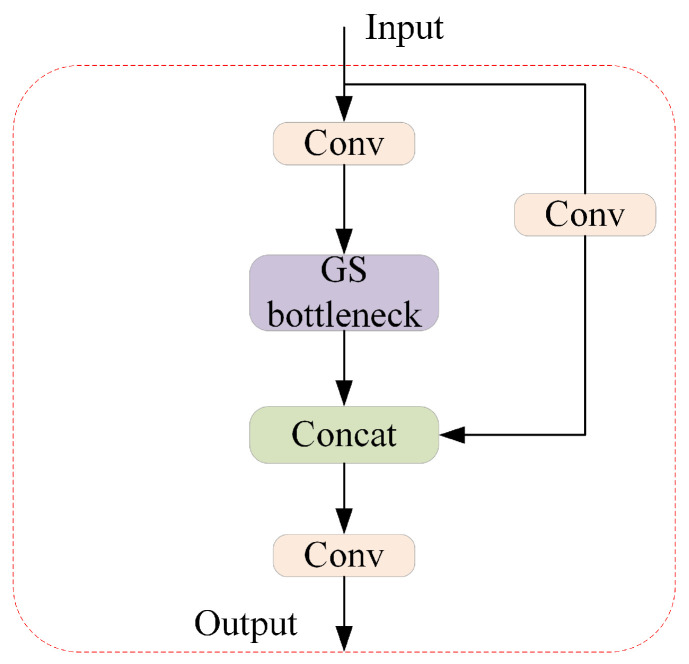
The VoV-GSCSP module.

The GSConv is introduced to solve the speed problem of predictive computing in Convolutional Neural Networks (CNNs). In the backbone network of CNNs, input images typically undergo a gradual spatial-to-channel transformation process. Each spatial compression and channel expansion of feature maps leads to a partial loss of semantic information. Channel-dense convolution (SC) maximally preserves implicit connections between each channel, whereas depthwise separable convolution (DSC) completely severs these connections. GSConv aims to maintain these connections as much as possible whilst reducing time complexity. Additionally, GSConv is used to design GS Bottleneck, as shown in Figure 9, which enhances feature non-linear expression and information reuse based on GSConv. As shown in Figure 10, the VoV-GSCSP module is a cross-stage partial network module designed by using a one-time aggregation method, which is employed to carry out effective information fusion between feature graphs of different stages.

**Figure 11:**
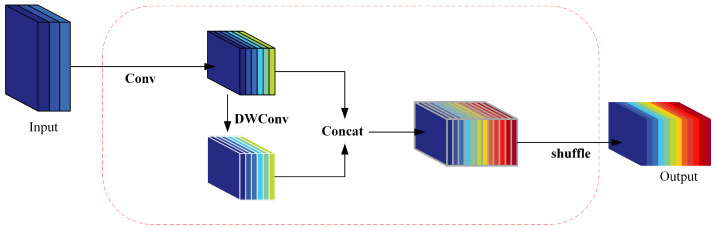
The GSConv module.

As seen in Figure 11, GSConv begins by performing downsampling with regular convolution on its input. Subsequently, it applies DWConv (depthwise convolution), concatenates the results of both convolutions and, finally, performs a shuffle operation to align corresponding channel numbers from the previous two convolutions together.

**Figure 12:**
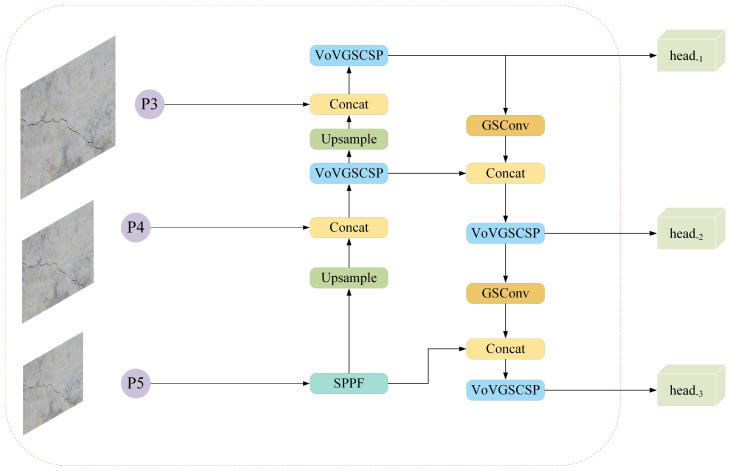
The new neck and head network in YOLOv8.

As shown in Figure 12, the new YOLOv8 neck and head networks replace Conv and C2f modules with GSConv and VoV-GSCSP modules, granting the model sufficient feature information to understand input data whilst reducing the model’s parameter number, achieving the purpose of lightweighting.

## 4. Dataset

This study used two datasets to train the model, including the RDD2022 dataset and the Wall Crack dataset.

RDD2022: Released by the University of Tokyo, it includes 47,420 road surface images collected from six countries: Japan, India, the Czech Republic, Norway, the United States and China. The dataset captures four types of concrete pavement crack damages, namely, D00 (longitudinal cracks), D10 (transverse cracks), D20 (mesh cracks) and D40 (potholes). Owing to the excessively large image sizes from Norway in comparison to other countries, they are deemed unsuitable for training. Therefore, in this paper, 16,648 photos from countries other than Norway are selected as the training set in the RDD2022 dataset.

Wall Crack: This dataset consists of 5882 concrete wall images collected from the Internet and literature, from which 6 different concrete surface damages can be captured, namely, D0 (exposed steel bars), D1 (weathering), D2 (cracks), D3 (delamination), D4 (spalling) and D5 (rust).

The RDD2022 dataset and the Wall Crack dataset are both used for detecting damage in concrete structures through image recognition. The RDD2022 dataset includes a large number of road crack images from different countries. The selection of the training set ensures a relatively balanced quantity of each type of damage and images from different countries. This allows the trained model to adequately represent different scenarios and types of damage, demonstrating excellent balance. The Wall Crack dataset consists of concrete wall images collected from the Internet and literature, capturing six different types of concrete surface damage. Although the number of images is relatively small, each type of damage is well-represented in the dataset, with a fairly balanced sample distribution. This ensures that the model can learn and recognize each type of damage fairly during training. Overall, these two datasets perform well in providing data support for concrete damage detection and also show excellent balance in data distribution.

## 5. Experiments and Results

### 5.1. Experimental Equipment and Evaluation Index

The primary development tool for this model is Python, with the open-source deep learning framework PyTorch as the network framework, accelerated by CUDA 11.8 for training. The hardware testing environment for this model includes an Intel^®^ Xeon^®^ Platinum 8375C CPU @ 2.90 GHz for the CPU and an NVIDIA RTX 4090 GPU with 24 GB of VRAM.

During training, the input images were set to 640 × 640, and SGD was used as the optimization function to train the model. The training consisted of 300 epochs with a batch size of 16 and an initial learning rate of 0.01. The experiment used the data augmentation algorithms identical to those used in the original YOLOv8 algorithm.

The evaluation indices used in this study included F1 score, mean Average Precision (mAP), GFLOPs and Frames Per Second (FPS). Precision and recall were fundamental indices, with F1 score and mAP, calculated on the bases of precision and recall, serving as the primary evaluation metrics to assess the model’s recognition accuracy. GFLOPs measured the complexity of the model or algorithm, and smaller GFLOPs indicated lower computational requirements and easier construction on low-end devices with reduced hardware performance demands. FPS refers to the number of frames detected per second, which depends not only on algorithm weights but also on the hardware configuration of the experimental equipment.

The precision ratio is the percentage of all results predicted correctly for a positive sample:

(6) $$Precision=\frac{TP}{TP+FP}$$

The recall rate is calculated on the basis of the proportion of all targets correctly predicted:

(7) $$Recall=\frac{TP}{TP+FN}$$

where TP is the number of correctly detected targets, FP is the number of falsely detected targets and FN is the number of missed targets among the correct targets.

The formula for calculating the average accuracy across n classes is as follows:

(8) $$mAP=\frac{1}{n}\sum_{i=1}^{n}\int_{0}^{1}Precision\left(Recall\right)d\left(Recall\right)$$

The *F*1 score comprehensively considers both precision and recall, providing a holistic reflection of the overall performance of the network. It is calculated as the harmonic mean of the two parameters as shown in the following:

(9) $$F1=2\frac{Precision\times Recall}{Precision+Recall}$$

### 5.2. Experimental Results and Analysis

#### 5.2.1. Comparison of Ablation Experiments

To validate the accuracy of the proposed algorithm improvements, experiments were conducted with several other models: YOLOv8n, YOLOv8n-L, YOLOv8n-G, YOLOv8n-GV, YOLOv8n-LG, YOLOv8n-LGV, YOLOv8n-GGV and YOLOv8-CD. Specifically, YOLOv8n-L incorporated the LSKA module before the SPPF module in the YOLOv8n backbone network, and YOLOv8n-G used the Ghost module within the YOLOv8n backbone network. YOLOv8n-GV introduced GSConv and VoV-GSCSP structures into the neck network of YOLOv8n. YOLOv8-CD is the proposed algorithm in this paper.

**Figure 13:**
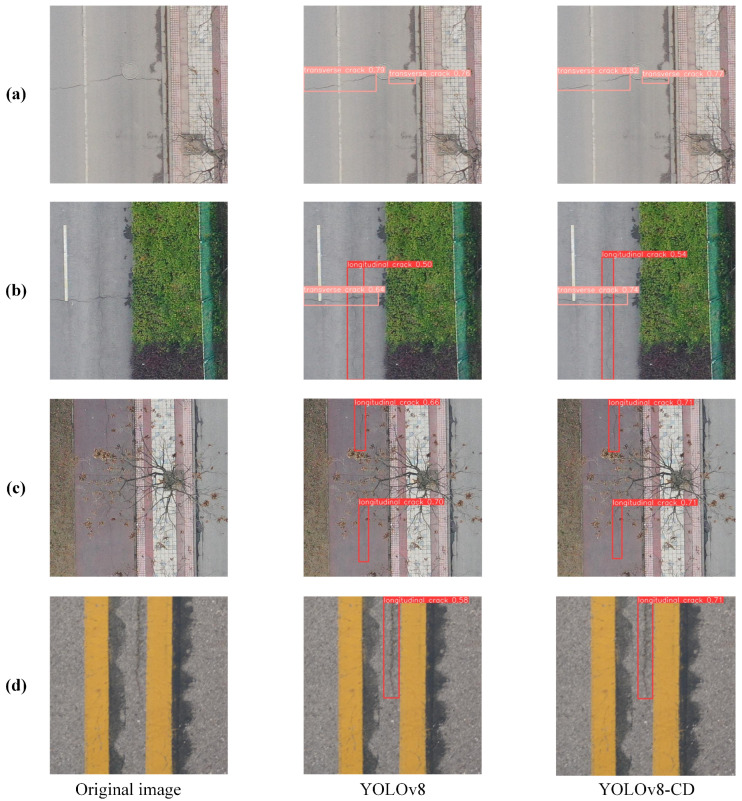
Detection results: (**a**) detection of transverse cracks; (**b**) detection of longitudinal cracks; (**c**) detection of multiple longitudinal cracks; (**d**) detection of cracks near road markings.

As shown in Table 1, the improved algorithm adopts an efficient network structure to improve the network structure of YOLOv8n, which improved the accuracy and reduced the calculation amount of the model. It also demonstrates that the Ghost module did not compromise the algorithm’s accuracy. Furthermore, the new head network increased accuracy whilst reducing model computational requirements. The introduced GSConv module only marginally increased the computational load. By integrating these improvements with the YOLOv8n model, it effectively reduced the deployment difficulty and cost on mobile terminals whilst remarkably enhancing accuracy in real-time scenarios. Figure 13 presents the detection performance on the RDD2022 dataset before and after improvements, showing the significant improvements in the detection accuracy of cracks in (a), (b), (c) and (d).

#### 5.2.2. Evaluation of Practical Application Detection

##### Application in the RDD2022 Dataset

**Figure 14:**
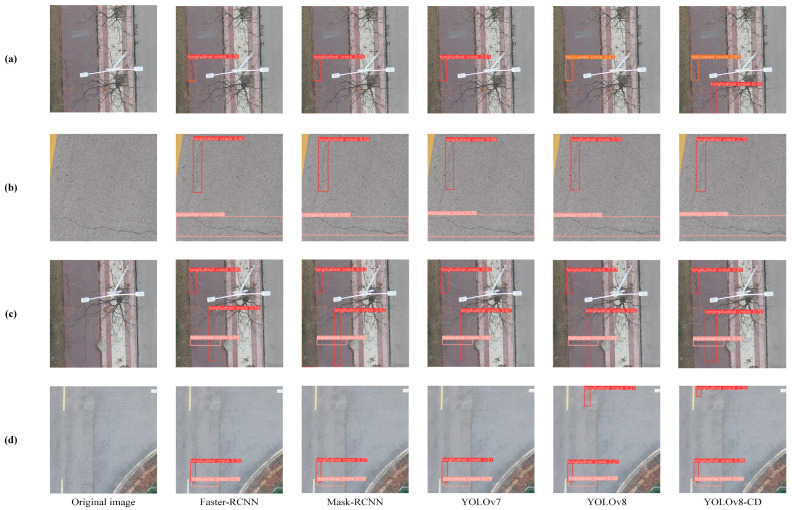
A comparison of detection results for different algorithms with the RDD2022 dataset: (**a**) detection of longitudinal cracks; (**b**) detection of transverse cracks; (**c**) detection of mixed cracks; (**d**) detection of cracks near road markings.

Figure 14 displays the detection results of the YOLOv8-CD algorithm in the RDD2022 dataset. The improved algorithm identified more crack targets than the original algorithm, with varying degrees of detection accuracy enhancement shown in the figures. Both (a) and (d) detected cracks that were missed by other algorithms. The results show that the improved YOLOv8-CD algorithm effectively detected concrete surface cracks, accurately identifying their positions and categories and demonstrating strong robustness and accuracy.

To further validate the detection performance of the model for different targets, Table 2 shows the performance of YOLOv8n and the improved model, YOLOv8-CD, under various conditions. The data show that YOLOv8-CD achieved higher detection accuracy in each category than YOLOv8n. Specifically, mAP50 and mAP50-90 improved by 15.2% and 22.7%, respectively.

To further validate the algorithm’s performance, this study compared YOLOv8-CD with other object detection algorithms using the RDD2022 dataset. Table 3 shows the results.

##### Application in the Wall Crack Dataset

**Figure 15:**
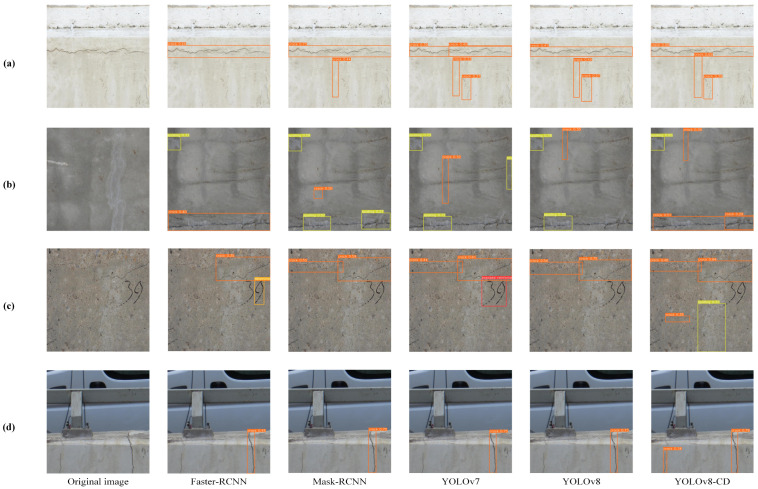
A comparison of detection results for different algorithms with the Wall Crack dataset: (**a**) detection of cracks on concrete walls; (**b**) detection of multiple small cracks on concrete walls; (**c**) detection of cracks on concrete walls with influencing factors; (**d**) detection of cracks in bridges.

The Wall Crack dataset was also tested in this study. Figure 15 shows the detection results of the YOLOv8-CD algorithm using the Wall Crack dataset. Remarkable improvements in crack detection accuracy were observed in Figure 15a,b, whereas Figure 15c,d demonstrated cracks detected by YOLOv8-CD that were missed by other algorithms. The results indicate that the optimized YOLOv8-CD algorithm performed excellently in detecting concrete surface crack objects, accurately locating and identifying various types of cracks and exhibiting high stability and reliability. Additionally, the algorithm showed outstanding performance in detecting other types of damage on concrete surfaces.

To further validate the model’s detection performance with different targets, Table 4 presents the performance of YOLOv8n and the improved model YOLOv8-CD under various conditions. The table shows that YOLOv8-CD achieved higher detection accuracy in each category than YOLOv8n. Specifically, mAP50 and mAP50-90 improved by 12.3% and 17.2%, respectively.

To further validate the algorithm’s performance, this study also compared YOLOv8-CD with other object detection algorithms using the Wall Crack dataset. Table 5 shows the results.

#### 5.2.3. Cross-Validation

To ensure the generalization ability and robustness of the model, this paper uses the Wall Crack dataset as an example, and a five-fold cross-validation method was employed in the experiments. Specifically, the entire dataset was randomly divided into five equal subsets. In each iteration, four subsets were used for model training, and the last subset for model validation. The process was repeated five times, each time selecting a different subset as the validation set, while the remaining four subsets were used for training. The final model performance was evaluated by averaging the results of the five validations.

The advantage of five-fold cross-validation is that it fully utilizes every sample in the dataset for both training and validation, thereby reducing the randomness caused by data partitioning and obtaining more reliable model performance evaluation metrics. In experiments, five-fold cross-validation not only effectively evaluated the model’s accuracy and recall rate, but also helped adjust the hyperparameters of the model and prevent the occurrence of overfitting. Table 6 shows the results of the five-fold cross-validation.

After cross-validation, it was found that the model’s performance was consistent across all folds, with an average precision of 91.5%, an average recall of 85.9%, an average mAP50 of 93.3%, and an average mAP50-95 of 77.6%. These results indicate that the model had high precision and recall, and it also demonstrated high average precision across different overlap thresholds. So, the model has good generalization ability and robustness.

## 6. Conclusions

Concrete surface crack detection is an important research area in structural safety, particularly in the structural assessment of roads, buildings and bridges. However, traditional crack detection methods typically rely on manual inspection, which is not only time-consuming and labor-intensive but also inefficient for meeting the demands of large-scale detection. Therefore, the automation of crack detection with deep learning algorithms has become a hotspot in related fields. Research have shown that traditional deep learning algorithms have been applied in crack detection. For example, Zhang et al. [34] proposed a deep learning model that was able to automatically detect and identify cracks from road images, which was validated in actual road crack detection. Zou et al. [35] proposed a DeepCrack model, which was also tested in various actual scenarios. Although these methods have been used in practice, their generalization ability and detection accuracy may be limited when dealing with the complexity and diversity of concrete surfaces.

As an advanced object detection algorithm, the YOLOv8 algorithm has shown a high potential in crack detection owing to its speed and high accuracy. However, there are numerous limitations when dealing with cracks on concrete surfaces. For instance, YOLOv8 may be disrupted by other textures, stains or shadows on concrete surfaces, leading to decreased accuracy in crack recognition. Therefore, this paper proposes an improved concrete surface crack detection algorithm based on YOLOv8n. The improved YOLOv8-CD algorithm of this paper is primarily applied to the crack detection on concrete surfaces. It can handle various types of cracks on concrete surfaces, including small and large cracks, while maintaining high detection accuracy and detection efficiency. This makes it suitable for large-scale crack detection tasks. This method is of great significance for the maintenance and repair of concrete structures. The main conclusions drawn are as follows:

(1) In the crack detection process of concrete surfaces, to better capture the unique characteristics of concrete surface cracks, this study innovatively combines the advantages of visual attention networks and large-scale convolutional attention, introducing a novel LSKA module. This module not only effectively extracts the entire feature information of concrete surface cracks but also focuses on the local details of cracks, particularly adapting to the characteristics of cracks that are prone to breakage, have large spans and are thin and long. Through the LSKA module, the model pays extra attention to the shape of cracks, ensuring accurate identification and localization of cracks in complex concrete textures and backgrounds, thereby improving the accuracy and efficiency of crack detection.
(2) The Ghost module is integrated into the backbone network of YOLOv8, aiming to efficiently extract and refine crucial information from raw features at minimal computational cost, largely enhancing the model’s feature extraction capability. The Ghost module reduces redundant computations by introducing a novel feature generation method. Accordingly, it not only effectively reduces the number of parameters and computation amount of the model, but also further improves its feature extraction capability and detection performance in complex scenarios whilst maintaining efficiency.
(3) In the neck network of YOLOv8, to enhance the efficiency and performance of the model, the GSConv structure is introduced to replace the traditional convolutional structure. Simultaneously, a novel VoV-GSCSP module is incorporated on the basis of the characteristics of the YOLOv8 framework. This module integrates the GSConv structure whilst maintaining the advantages of the original network, allowing the effective utilization of computational resources during feature channel fusion and reducing floating-point operations. By introducing the GSConv and VoV-GSCSP modules, significant optimization is achieved in the neck network of YOLOv8 for feature extraction and fusion. This optimization enables the model to operate efficiently and accurately when dealing with complex images and detection tasks.
(4) This paper achieves efficient and accurate crack detection by improving the YOLOv8 algorithm. The improved algorithm has achieved notable performance improvement with multiple datasets, thus proving its effectiveness and practicability in the field of crack detection. Compared with the existing models, this method has higher detection accuracy whilst reducing demands on platform computing and storage capabilities, and deploying on resource-limited devices becomes easy. In the future, we will continue exploring other advanced deep learning algorithms and technologies to further enhance the accuracy and efficiency of crack detection. Additionally, we aim to deploy the improved model on resource-limited embedded detection devices to refine the proposed algorithm in practical applications.

## Figures and Tables

**Table 1:** Ablation experiment results.

Algorithm	LSKA	Ghost	GSConv	F1/%	mAP50/%	mAP50-95/%	GFLOPs	FPS
YOLOv8n				78.5	78.3	48.7	8.2	89
YOLOv8n-L	√			93.2	93.5	70.1	8.2	87
YOLOv8n-G		√		89.7	90.0	65.8	8.2	83
YOLOv8n-GV			√	89.2	89.6	65.1	8.3	79
YOLOv8n-LG	√	√		89.3	89.7	65.5	8.2	82
YOLOv8n-LGV	√		√	90.1	90.3	67.2	8.3	83
YOLOv8n-GGV		√	√	92.1	93.2	70.0	8.0	84
YOLOv8-CD	√	√	√	93.2	93.5	71.4	7.9	88

**Table 2:** A comparison of detection results for different types of cracks with the RDD2022 dataset.

Algorithm	Type	mAP50/%	mAP50-95/%
YOLOv8n	D00	74.4	47.8
	D10	75.0	43.1
	D20	91.0	64.6
	D40	72.7	39.5
	ALL	78.3	48.7
YOLOv8-CD	D00	89.0	67.1
	D10	93.3	67.4
	D20	99.1	85.9
	D40	92.8	65.4
	ALL	93.5	71.4

**Table 3:** Results comparison of different algorithms with the RDD2022 dataset.

Algorithm	F1/%	mAP50/%	mAP50-95/%	GFLOPs	FPS
Faster-RCNN	49.4	51.2	22.5	370.2	21
Mask-RCNN	56.4	54.8	25.0	110.6	24
YOLOv7	57.8	57.9	25.6	13.2	114
YOLOv8	78.4	78.3	48.7	8.2	89
YOLOv8-CD	93.2	93.5	71.4	7.9	88

**Table 4:** A comparison of detection results for different types of cracks with the Wall Crack dataset.

Algorithm	Type	mAP50/%	mAP50-95/%
YOLOv8n	D0	89.7	70.9
	D1	77.1	56.8
	D2	78.7	57.7
	D3	85.8	68.3
	D4	73.1	59.2
	D5	84.3	57.8
	ALL	81.5	61.8
YOLOv8-CD	D0	95.6	82.6
	D1	91.8	75.2
	D2	92.1	76.1
	D3	94.5	82.3
	D4	91.2	79.4
	D5	97.7	78.1
	ALL	93.8	79.0

**Table 5:** Results comparison of different algorithms with the Wall Crack dataset.

Algorithm	F1/%	mAP50/%	mAP50-95/%	GFLOPs	FPS
Faster-RCNN	49.6	51.0	21.5	370.2	21
Mask-RCNN	55.3	55.6	27.3	110.6	24
YOLOv7	56.2	57.9	25.6	13.2	114
YOLOv8	81.8	81.5	61.8	8.2	89
YOLOv8-CD	93.2	93.8	79.0	7.9	88

**Table 6:** The results of the cross-validation.

Fold	Training Set	Validation Set	Precision/%	Recall/%	mAP50/%	mAP50-95/%
1	Fold 2, 3, 4, 5	Fold 1	91.1	86.4	93.3	77.8
2	Fold 1, 3, 4, 5	Fold 2	91.4	86.0	92.8	77.2
3	Fold 1, 2, 4, 5	Fold 3	91.8	85.8	93.7	78.0
4	Fold 1, 2, 3, 5	Fold 4	91.2	85.3	93.1	77.4
5	Fold 1, 2, 3, 4	Fold 5	92.1	86.1	93.6	77.8
Average			91.5	85.9	93.3	77.6

## Data Availability

The data that support the findings of this study are available from the corresponding author upon reasonable request. The data are not publicly available due to privacy.
